# Vascular Cellular Adhesion Molecule-1 (VCAM-1) Expression in Mice Retinal Vessels Is Affected by Both Hyperglycemia and Hyperlipidemia

**DOI:** 10.1371/journal.pone.0012699

**Published:** 2010-09-13

**Authors:** Carin Gustavsson, Carl-David Agardh, Anna V. Zetterqvist, Jan Nilsson, Elisabet Agardh, Maria F. Gomez

**Affiliations:** Department of Clinical Sciences, Lund University, Malmö, Sweden; INSERM, France

## Abstract

**Background:**

Inflammation has been proposed to be important in the pathogenesis of diabetic retinopathy. An early feature of inflammation is the release of cytokines leading to increased expression of endothelial activation markers such as vascular cellular adhesion molecule-1 (VCAM-1). Here we investigated the impact of diabetes and dyslipidemia on VCAM-1 expression in mouse retinal vessels, as well as the potential role of tumor necrosis factor-α (TNFα).

**Methodology/Principal Findings:**

Expression of VCAM-1 was examined by confocal immunofluorescence microscopy in vessels of wild type (wt), hyperlipidemic (ApoE^−/−^) and TNFα deficient (TNFα^−/−^, ApoE^−/−^/TNFα^−/−^) mice. Eight weeks of streptozotocin-induced diabetes resulted in increased VCAM-1 in wt mice, predominantly in small vessels (<10 µm). Diabetic wt mice had higher total retinal *TNFα*, *IL-6* and *IL-1β* mRNA than controls; as well as higher soluble VCAM-1 (sVCAM-1) in plasma. Lack of TNFα increased higher basal VCAM-1 protein and sVCAM-1, but failed to up-regulate *IL-6* and *IL-1β* mRNA and VCAM-1 protein in response to diabetes. Basal VCAM-1 expression was higher in ApoE^−/−^ than in wt mice and both *VCAM-1* mRNA and protein levels were further increased by high fat diet. These changes correlated to plasma cholesterol, LDL- and HDL-cholesterol, but not to triglycerides levels. Diabetes, despite further increasing plasma cholesterol in ApoE^−/−^ mice, had no effects on VCAM-1 protein expression or on sVCAM-1. However, it increased *ICAM-1* mRNA expression in retinal vessels, which correlated to plasma triglycerides.

**Conclusions/Significance:**

Hyperglycemia triggers an inflammatory response in the retina of normolipidemic mice and up-regulation of VCAM-1 in retinal vessels. Hypercholesterolemia effectively promotes VCAM-1 expression without evident stimulation of inflammation. Diabetes-induced endothelial activation in ApoE^−/−^ mice seems driven by elevated plasma triglycerides but not by cholesterol. Results also suggest a complex role for TNFα in the regulation of VCAM-1 expression, being protective under basal conditions but pro-inflammatory in response to diabetes.

## Introduction

The pathogenesis of diabetic retinopathy has lately been recognized to involve low-grade, chronic inflammation[1], [2], [3], proposed to be the result of persistent hyperglycemia as well as of dyslipidemia[1], [4], [5]. Up-regulation of inflammatory mediators and adhesion molecules are early features of diabetic retinopathy[6], leading to accumulation of leukocytes, altered vessel reactivity and subsequent activation of receptors and transcription factors, ultimately resulting in apoptosis or proliferation of various cell types in the retina[5], [7], e.g., loss of pericytes and proliferation of endothelial cells. However, it is still a matter of debate whether the inflammatory response is a local phenomenon or not, since clinical studies show variable associations between markers of systemic inflammation and risk of diabetic retinopathy[8], [9], [10].

Endothelial cells release multiple inflammatory mediators and express various adhesion molecules such as intercellular and vascular cellular adhesion molecules (ICAM-1, VCAM-1), P- and E-selectins[4]. These are membrane proteins necessary for anchoring leukocytes to the vessel wall and are well established markers of endothelial dysfunction in inflammatory conditions such as atherosclerosis[7], [11], [12]. Soluble forms of these adhesion molecules and selectins have been demonstrated in serum of diabetic patients, suggesting that they may play a role in diabetic endothelial activation[6], [13], [14]. Moreover, increased levels of soluble (s)VCAM-1 have been demonstrated in the vitreous of diabetic patients[15], [16], [17], [18]. In type 2 diabetic subjects (T2D), serum levels of sVCAM-1 and sE-selectin are increased both in patients with micro- and macrovascular complications, whereas sICAM-1 levels are higher only in the microvascular group[19]. This suggests potential differential regulation of adhesion molecules and maybe also differential functions. In line with this idea, recent studies have shown associations between sVCAM-1 in human serum and proliferative diabetic retinopathy, but not for sICAM-1[20] [8]. ICAM-1 has been widely used as a marker of endothelial activation in experimental studies of diabetic retinopathy, but much less is known about VCAM-1 in this context. Accordingly, the first aim of the present study was to evaluate potential changes in VCAM-1 expression in a streptozotocin (STZ) model of diabetes in mouse.

Dyslipidemia is a well established proinflammatory agent in large arterial vessel disease[21] and could be of importance in the pathogenesis of microvascular complications of diabetes[22]. In the Diabetes Control and Complications Trial (DCCT)[23], it was shown that severity of retinopathy was associated with increasing serum triglycerides and inversely associated with HDL-cholesterol levels[24]. There is also evidence for the involvement of hypercholesterolemia in the formation of hard exudates in diabetic retina, with potential negative effects on disease progression[25]. Lipid-modifying fenofibrate has been shown to reduce the need for laser treatment of sight-threatening diabetic retinopathy, but the effect did not seem to be attributable to changes in lipid profile[26]. Furthermore, results from the ACCORD Study Group and ACCORD Eye Study Group showed that combination therapy (simvastatin plus fenofibrate) reduced the rate of progression of diabetic retinopathy[27]. Despite accumulating clinical evidence, the underlying mechanisms of lipid involvement are not clear and experimental data are sparse. In the present study, we used the genetically modified apolipoprotein E deficient (ApoE^−/−^) mouse, a widely used mouse model of atherosclerosis and natural hypercholesterolemia, to study the effect of dyslipidemia on endothelial VCAM-1 expression. ApoE is a structural component of astrocytes in the central nervous system and of Müller cells in the retina, and it has important lipid transport regulatory and immunologic functions[28]. In ophthalmology, the ApoE^−/−^ model is most frequently used in neovascular age-related macular degeneration (AMD) experiments, but is not as widely used for studies of diabetic retinopathy. Although the lipid profile of the ApoE^−/−^ differs somehow from that of dyslipidemic human subjects, we suggest that this model may be relevant for addressing the issue of inflammation and/or endothelial activation in diabetic retinopathy. The second aim of our study was thus to assess whether the VCAM-1 expression pattern in retinal vessels was different in dyslipidemic compared to wild type (wt) mouse, and how diabetes would influence such an expression. As a complement to genetically caused dyslipidemia, we also explored the effects of high fat diet on VCAM-1 expression in retinal vessels.

There is strong evidence that tumor necrosis factor-α (TNFα) is involved in inflammatory processes in diabetic retinopathy[4], [29]. TNFα is one of the key cytokines in inflammation, but the pathways directly or indirectly activated upon TNFα engagement may vary widely and lead to different outcomes depending on cell- and receptor type as well as on environmental factors[30]. Both inflammatory and anti-inflammatory TNFα actions have been described[31], [32]. The third aim of the present study was to evaluate the influence of TNFα on endothelial VCAM-1 expression in diabetes and/or dyslipidemia, using TNFα knockout mice.

## Results

### Effect of diabetes and high fat diet on body weight, blood glucose, triglycerides and cholesterol

To investigate the effects of diabetes on VCAM-1 expression, as well as the potential role of TNFα, C57BL/6 wild-type (wt), ApoE^−/−^, TNFα^−/−^ and ApoE^−/−^/TNFα^−/−^ mice were chow-fed until 22 weeks of age, injected with STZ or vehicle once a day for 5 days and kept on chow diet for additional 8 weeks. Mean body weight, blood glucose, plasma triglycerides, total cholesterol as well as LDL- and HDL-cholesterol for the different genotypes are listed in **Table 1**. STZ treatment significantly increased blood glucose values by at least 2-fold in all groups. At the time mice were euthanized, diabetic mice had lower body weight than non-diabetic littermates. As expected, ApoE^−/−^ mice had higher plasma triglycerides, total cholesterol and LDL-cholesterol than wt mice and as also described by others[33], [34], all three parameters were further increased in diabetic ApoE^−/−^ mice. In ApoE^−/−^ mice also deficient for TNFα, cholesterol levels were also increased by diabetes and this effect was more pronounced than in ApoE^−/−^ mice. Hence, diabetic ApoE^−/−^ and ApoE^−/−^/TNFα^−/−^ mice had both hyperglycemia and hyperlipidemia, whereas wt and TNFα^−/−^ mice exhibited hyperglycemia but no significant changes in plasma lipids.

**Table 1:** Body weight, blood glucose, triglycerides, total cholesterol, LDL and HDL in control and diabetic mice of different genotypes (experimental set 1).

Genotype	Body weight (g)	Blood glucose (mmol/l)	Triglycerides (mmol/l)	Total cholesterol (mmol/l)	HDL cholesterol (mmol/l)	LDL cholesterol (mmol/l)
wt control (n = 12)	23.4±1.5	7.1±0.6	0.39±0.09	1.34±0.19	0.78±0.28	0.43±0.23
wt diabetes (n = 16)	20.5±1.9 ***	16.9±5.9 ***	0.55±0.15	1.70±0.31	1.02±0.42	0.49±0.36
ApoE^−/−^ control (n = 24)	23.6±2.4	8.8±0.8	0.62±0.17#	9.08±2.09###	0.51±0.20	7.17±2.05###
ApoE^−/−^ diabetes (n = 29)	21.3±1.8 ***	18.6±5.4 ***	0.89±0.36 ***	14.10±4.97 ***	0.41±0.11	12.41±5.14 ***
TNF-α^−/−^ control (n = 10)	23.4±1.0	7.1±1.0	0.44±0.10	1.54±0.29		
TNF-α^−/−^ diabetes (n = 14)	20.6±0.5 **	14.4±4.3 ***	0.41±0.16	2.17±0.53		
ApoE^−/−^/TNF-α^−/−^ control (n = 15)	22.7±1.1	7.7±1.0	0.57±0.12	9.78±1.88		
ApoE^−/−^/TNF-α^−/−^ diabetes (n = 15)	20.0±2.7 ***	21.0±5.9 ***	0.72±0.23	20.34±5.04 ***		

Values represent mean ± SD. HDL, high density lipoprotein; LDL, low density lipoprotein. Blood glucose represents average blood glucose values during the experiments (from week 2 until termination). Body weight and lipids express values measured at the end of the experiments. Two HDL-cholesterol measurements in the “wt diabetes” group yielded higher than total cholesterol values, and were therefore excluded from calculations. Missing values are due to limited plasma availability. Two-way analyses of variance (for the effects of diabetes and genotype) revealed no interactions between factors. Bonferroni posttests yielded

**p<0.01,

***p<0.001 for comparisons between control and diabetes of the same genotype;

# p<0.05 and

### p<0.001 vs. wt control.

To further elucidate the impact of hyperlipidemia on endothelial activation, two separate sets of experiments were performed, a first one in which hyperlipidemic ApoE^−/−^ mice were fed a high fat diet (HFD) for 4 weeks and a second one in which normolipidemic mice were fed a HFD for 4 or 8 weeks. Mean body weight, blood glucose and plasma lipids for these experiments are summarized in **Tables 2** and **3**.

**Table 2:** Body weight, blood glucose, triglycerides, total cholesterol, LDL and HDL in wt and ApoE^−/−^ mice, fed normal chow or HFD (experimental set 2).

Genotype	Body weight (g)	Blood glucose (mmol/l)	Triglycerides (mmol/l)	Total cholesterol (mmol/l)	HDL cholesterol (mmol/l)	LDL cholesterol (mmol/l)
wt chow diet (n = 7)	24.0±4.9	9.3±2.9	0.63±0.41	1.76±0.48	0.71±0.23	0.76±0.35
ApoE^−/−^ chow diet (n = 5)	23.6±1.7	9.4±0.6	0.47±0.11	10.35±5.19 **	0.20±0.08 ***	6.77±1.10 **
ApoE^−/−^ HFD (n = 5)	22.5±1.0	8.1±0.8	0.62±0.12	8.81±4.72 *	0.27±0.12 **	13.11±5.48 *** #

Values represent mean ± SD. HFD, high fat diet; HDL, high density lipoprotein; LDL, low density lipoprotein. HDL/LDL data are missing for one of the ApoE^−/−^ animals fed with HFD, since the HDL value was below detection limit. One-way analyses of variance followed by Bonferroni tests were performed;

*p<0.05,

**p<0.01,

***p<0.001 vs. wt chow diet;

# p<0.05 vs. ApoE^−/−^ chow diet.

**Table 3:** Body weight, blood glucose, triglycerides and total cholesterol in FVBN mice, fed normal chow or HFD for 4 or 8 weeks (experimental set 3).

Genotype	Body weight (g)	Blood glucose (mmol/l)	Triglycerides (mmol/l)	Total cholesterol (mmol/l)
FVBN chow diet (n = 16)	34.9±4.7	9.5±1.1	1.23±0.47	3.33±0.52
FVBN HFD 4 weeks (n = 5)	40.8±6.2	12.6±3.4 *	0.65±0.22 *	5.17±1.44 ***
FVBN HFD 8 weeks (n = 8)	41.8±7.2 *	11.3±2.6	0.95±0.45	5.95±0.95 ***

Values represent mean ± SD. HFD, high fat diet. One-way analyses of variance followed by Bonferroni tests were performed;

*p<0.05,

***p<0.001 vs. wt chow diet.

### Effect of diabetes on VCAM-1 protein expression in retinal vessels

**Figure 1:**
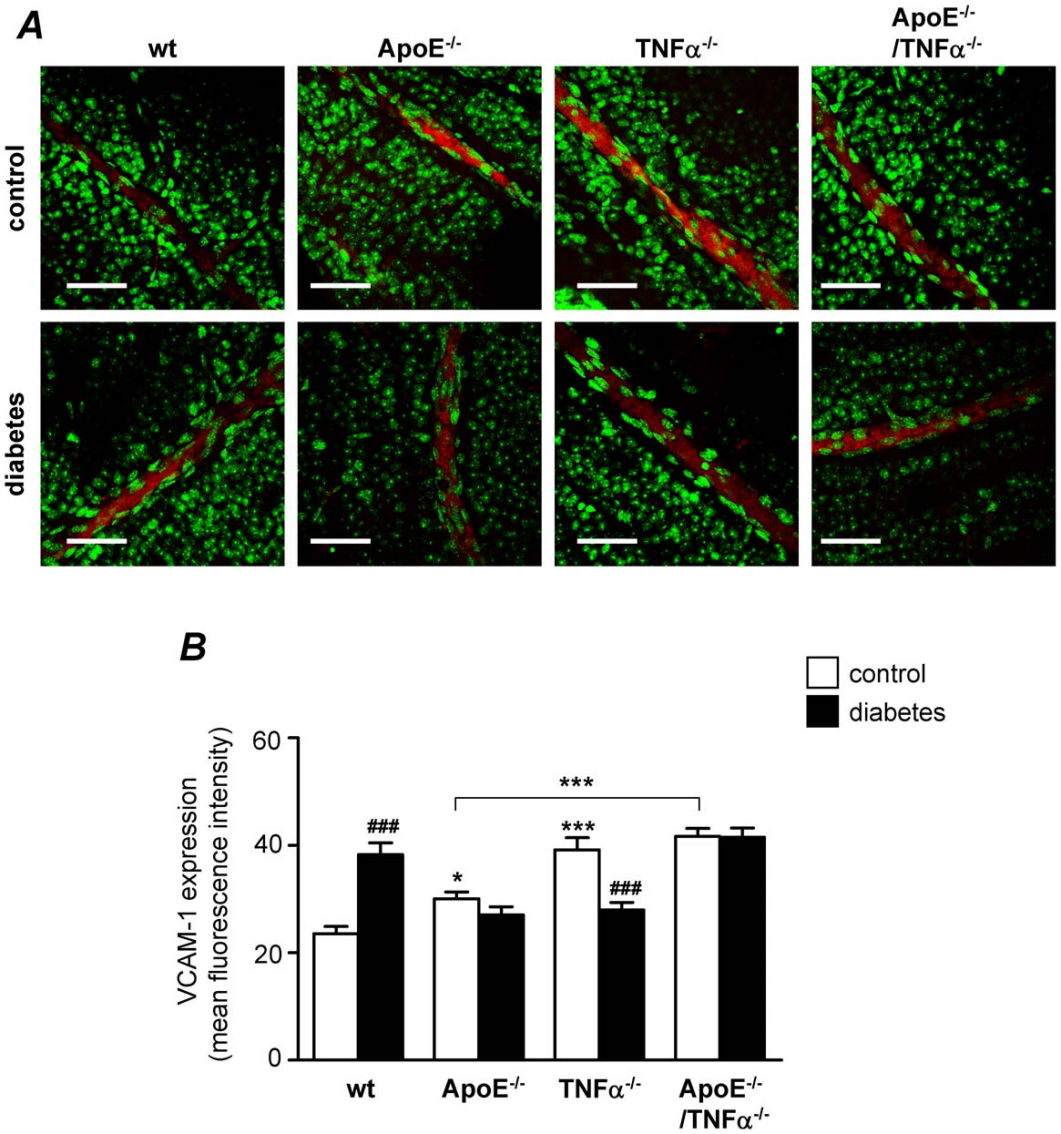
Effects of diabetes on VCAM-1 protein expression in retinal vessels. (**A**) Confocal immunofluorescence microscopy images showing VCAM-1 staining (red) in retinal whole-mounts from control non-diabetic and diabetic wild type (wt), ApoE^−/−^, TNFα^−/−^ and ApoE^−/−^/TNFα^−/−^ mice. The DNA-binding dye SYTOX (green) was used for nuclear localization. Bars = 50 µm. Measurements were performed 8 weeks after the first STZ-injection, when mice were 30 weeks of age. (**B**) Summarized data from experiments in A showing mean fluorescence intensity of VCAM-1 in the different groups. White bars represent control and black bars diabetic mice. Two-way analysis of variance (for the effects of diabetes and genotype) revealed significant interactions between factors. Bonferroni posttests yielded *p<0.05 and ***p<0.001 for comparisons between ApoE^−/−^ and TNFα^−/−^, respectively vs. wt control; ***p<0.001 for comparison between ApoE^−/−^ and ApoE^−/−^/TNFα^−/−^; and ^###^ *p*<0.001 for comparisons between control and diabetes of the same genotype.

To determine the effect of diabetes on endothelial activation in retinal arteries, we measured VCAM-1 expression by confocal immunofluorescence microscopy. As previously shown by us[35] and others[36], VCAM-1 was detected in both endothelial and smooth muscle cells in arteries from mouse, with higher expression in endothelial cells. As shown in **Figure 1**, when non-diabetic mice of different genotypes were compared, ApoE^−/−^ mice were found to express higher VCAM-1 levels than wt mice (*p*<0.05), suggesting that hyperlipidemia may induce VCAM-1 expression in retinal vessels. Interestingly, TNFα deficient mice also expressed higher VCAM-1 levels than wt mice (*p*<0.001) and TNFα deficient ApoE^−/−^ mice exhibited higher VCAM-1 expression than ApoE^−/−^ mice (*p*<0.001), suggesting that TNFα may be involved in the regulation of basal VCAM-1 expression.

**Figure 2:**
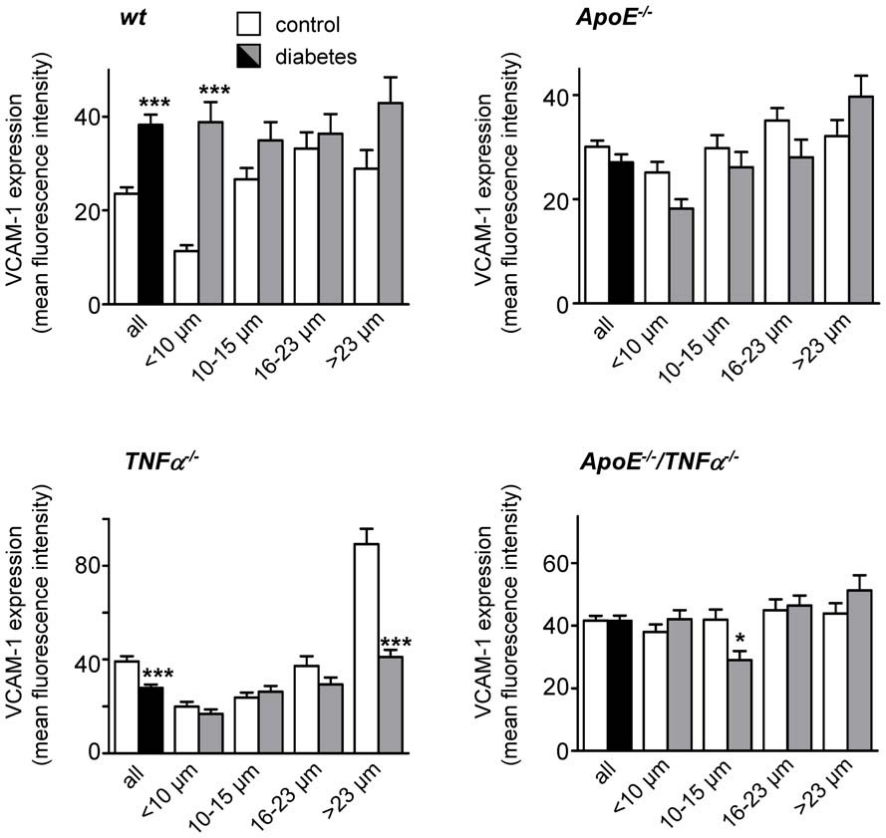
The effect of diabetes on VCAM-1 expression is dependent on retinal vessel size. Summarized data from confocal immunofluorescence microscopy experiments showing mean fluorescence intensity of VCAM-1 in retinal vessels divided into quartiles depending on vessel diameter (<10 µm, 10–15 µm, 16–23 µm and >23 µm). Each graph represents the expression-diameter relationship in the different genotypes. White bars are for control and grey bars for diabetes. The two first bars in each graph (in white and black, for control and diabetes respectively) show the same data as in **Figure 1**, but are displayed here as references. *p<0.05 and ***p<0.001 indicate differences between control and diabetes of the same size and genotype.

When the effect of diabetes was evaluated (**Figure 1**), VCAM-1 expression was found to be increased in wt mice (*p*<0.001), but no effects were demonstrated in hyperlipidemic ApoE^−/−^ mice. VCAM-1 expression was even decreased in diabetic TNFα^−/−^ mice when compared to non-diabetic mice of the same genotype (*p*<0.001). The level of VCAM-1 expression correlated to the vessel diameter, with higher expression in larger vessels. Correlations were significant for all genotypes (*r* = 0.264 and *r* = 0.616 for wt and TNFα^−/−^ mice, respectively; *p*<0.001), with weaker correlations for ApoE^−/−^ and ApoE^−/−^/TNFα^−/−^ mice (*r* = 0.124 and *r* = 0.123, respectively; *p*<0.01). When vessels were divided in quartiles according to vessel diameter (<10 µm, 10–15 µm, 16–23 µm and >23 µm; **Figure 2**), it became evident that the overall effect of diabetes on VCAM-1 expression in wt mice was predominantly driven by changes in the very small vessels (<10 µm), whereas the overall effect observed in TNFα^−/−^ mice was predominantly driven by changes in the larger vessels (>23 µm).

Within the same retina, not all arteries expressed VCAM-1 protein. To assess whether genotype or diabetes had any impact on the pattern of VCAM-1 expression, the number of VCAM-1 positive vessels in each retina was calculated as percentage of total number of vessels. A vessel was considered positive if mean VCAM-1 intensity was higher than mean background intensity, whereas it was considered negative if mean VCAM-1 intensity was below. The average background intensity did not differ between genotypes. The percentage of VCAM-1 positive vessels in wt and ApoE^−/−^ mice was lower in diabetic animals (*p*<0.05 and *p*<0.001, respectively), whereas no differences were observed in the TNFα deficient genotypes (**Figure S1**).

### Effect of diabetes on endothelial activation

**Figure 3:**
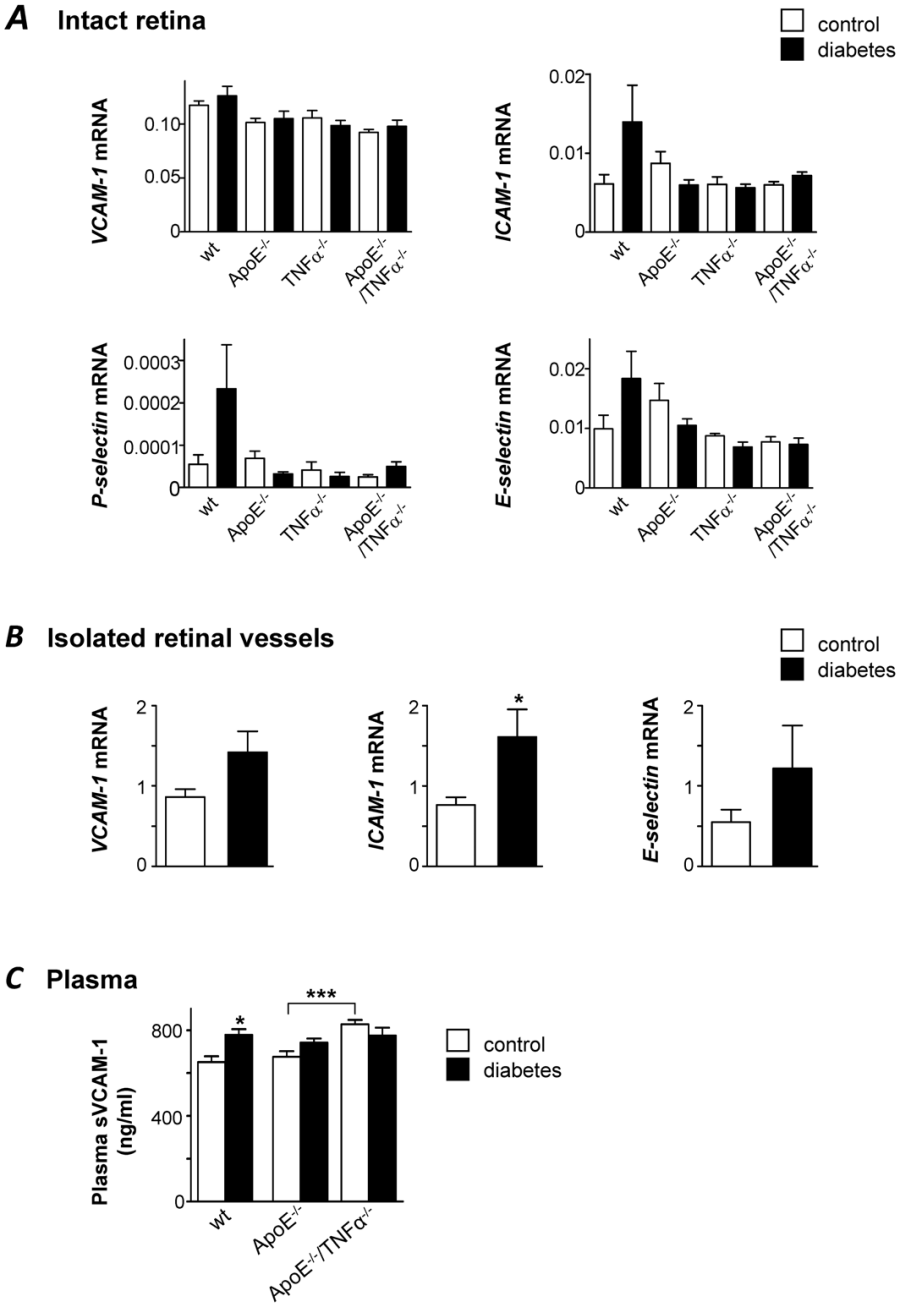
Changes in the expression of vascular adhesion markers in retinal vessels are difficult to resolve if measured in total retinal RNA. (**A**) Expression of *VCAM-1*, *ICAM-1*, *P-* and *E-selectin* mRNA was measured by real time RT-PCR in intact retinas of control (white bars) and diabetic (black bars) wt, ApoE^−/−^, TNFα^−/−^ and ApoE^−/−^/TNFα^−/−^ mice. No statistically significant differences were seen between genotypes or between retinas from control and diabetic mice.Results are normalized to the expression of the housekeeping control cyclophilin B. (**B**) *VCAM-1*, *ICAM-1* and *E-selectin* mRNA were measured in isolated retinal vessels from control (white) and diabetic (black) ApoE^−/−^ mice. Values are normalized to the expression of cyclophilin B and GAPDH. *p<0.05 indicates difference to the control group. (**C**) Mean sVCAM-1 concentrations in plasma (ng/ml), measured by ELISA are depicted for control (white bars) and diabetic (black bars) wt, ApoE^−/−^ and ApoE^−/−^/TNFα^−/−^ mice. *p<0.05 vs. wt control and ***p<0.001 for differences between non-diabetic ApoE^−/−^ and ApoE^−/−^/TNFα^−/−^ mice. For **A–C**, measurements were performed 8 weeks after the first STZ-injection, when mice were 30 weeks of age.

As a complementary approach to evaluate a potential endothelial dysfunction in response to diabetes, the expression of *VCAM-1*, *ICAM-1*, *P-selectin* and *E-selectin* mRNA was measured in intact retinas of wt, ApoE^−/−^, TNFα^−/−^ and ApoE^−/−^/TNFα^−/−^ mice by real time RT-PCR. As opposed to what we found when VCAM-1 protein was measured in retinal arteries by confocal microscopy (see **Figure 1**), no differences in *VCAM-1* mRNA expression were observed between genotypes or in response to diabetes when whole retinas were examined (**Figure 3A**). For the other adhesion molecules *ICAM-1*, *P-selectin* and *E-selectin*, mRNA levels were higher in diabetic wt mice compared to non-diabetic controls of the same genotype, but differences did not reach statistical significance. Lack of ApoE or TNFα had no impact on basal expression of these adhesion molecules. Further, diabetes had no effect on the mRNA levels of any of these targets in ApoE or TNFα deficient mice (**Figure 3A**). However, when mRNA levels of *VCAM-1*, *ICAM-1* and *E-selectin* were studied in isolated retinal vessels from ApoE^−/−^ using the same assays as for intact retina, a clear increase of *ICAM-1* in response to diabetes was observed (*p*<0.05) and similar trends were seen for *VCAM-1* and *E-selectin* (n.s.; **Figure 3B**). Even though blood glucose, plasma triglycerides and total cholesterol were all significantly increased by diabetes, expression of endothelial adhesion molecules correlated only to triglyceride levels. Correlation parameters were *r* = 0.429 for *VCAM-1* (*p*<0.05), *r* = 0.441 for *ICAM-1* (*p*<0.05) and *r* = 0.480 for *E-selectin* (*p*<0.05). These results underscore the importance of measuring expression of vascular endothelial markers on isolated vessels and the limited resolution of expression measurements in whole retina homogenates.

For a more systemic indication of endothelial activation in response to diabetes, we measured soluble VCAM-1 (sVCAM-1) in plasma from wt, ApoE^−/−^ and ApoE^−/−^/TNFα^−/−^ mice. As shown in **Figure 3C**, differences in sVCAM-1 resemble the pattern of expression of VCAM-1 protein observed in retinal vessels by confocal microscopy (see **Figure 1**). TNFα deficient ApoE^−/−^ mice exhibited higher sVCAM-1 levels than ApoE^−/−^ mice (*p*<0.001), again supporting a role for TNFα in the regulation of basal VCAM-1 expression. Also, sVCAM-1 levels were increased in wt mice in response to diabetes (*p*<0.05), but no effect was observed in hyperlipidemic ApoE^−/−^ mice (**Figure 3C**).

**Figure 4:**
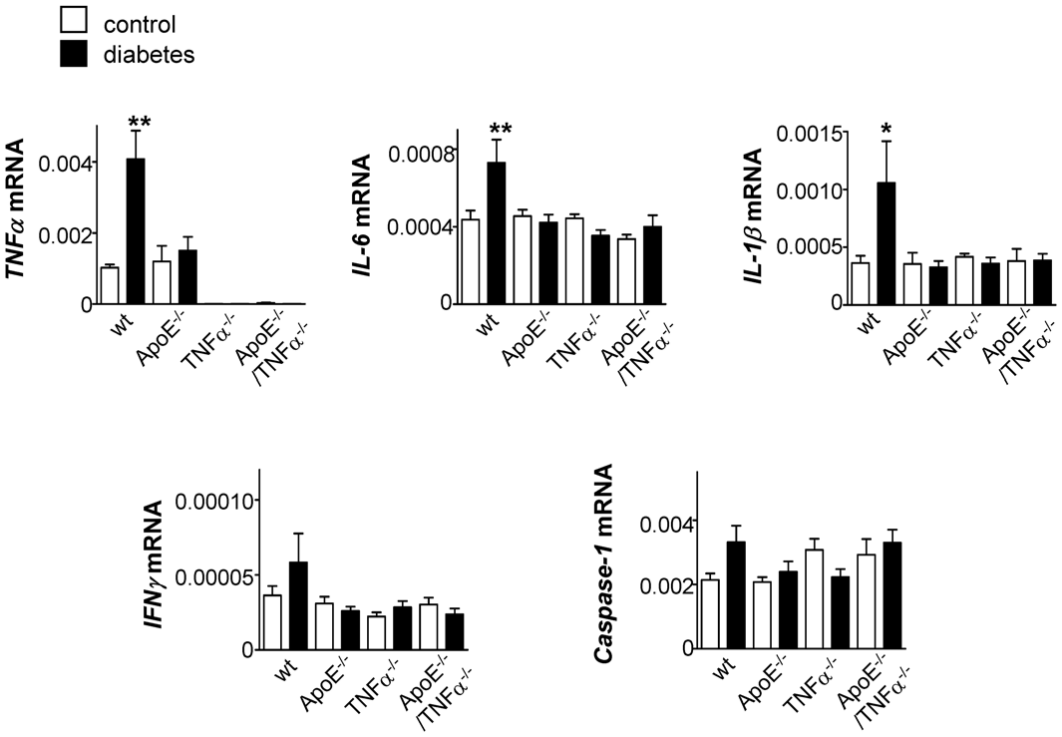
Diabetes enhances the expression of inflammatory cytokines in the retina of wt mice but not of ApoE- or TNFα deficient mice. Expression of *TNF*α, *IL-6*, *IL-1β*, *IFNγ* and *caspase-1* mRNA was measured by real time RT-PCR in intact retinas of control (white bars) and diabetic (black bars) wt, ApoE^−/−^, TNFα^−/−^ and ApoE^−/−^/TNFα^−/−^ mice. Values are normalized to the expression of cyclophilin B. *p<0.05 and **p<0.01 indicates differences to control wt mice.

The effect of diabetes on the expression of various inflammatory (TNFα, IL-6, IL-1β and IFNγ) and apoptosis markers (caspase-1) was also evaluated in intact retinas from wt, ApoE^−/−^, TNFα^−/−^ and ApoE^−/−^/TNFα^−/−^ mice. In wt mice, diabetes resulted in a clear increase in *TNF*α, *IL-6* and *IL-1β* mRNA expression levels (p<0.01, p<0.01 and p<0.05, respectively) and in a tendency to higher *IFNγ* and *caspase-1* mRNA levels (n.s.; **Figure 4**). No significant changes were detected in retinas of ApoE or TNFα deficient mice in response to diabetes. When expression levels were compared among mice of different genotypes, lack of *TNF*α mRNA was confirmed in TNFα^−/−^ and ApoE^−/−^/TNFα^−/−^ animals, but no significant differences in the basal levels of the other studied targets could be dissected using RNA from whole retinas (**Figure 4**).

### Effect of hyperlipidemia on VCAM-1 expression in retinal vessels

**Figure 5:**
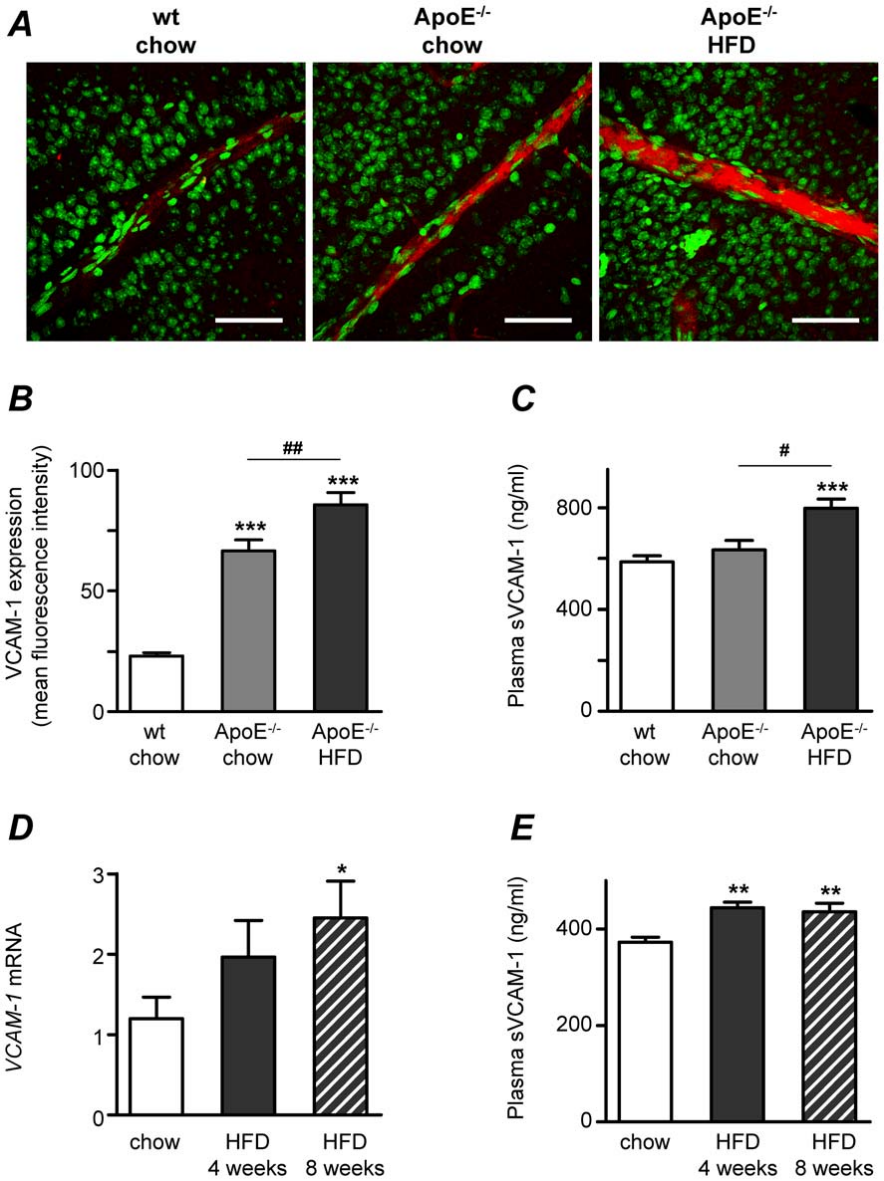
High fat diet (HFD) increases VCAM-1 expression in retinal vessels. (**A**) Confocal immunofluorescence microscopy images showing VCAM-1 expression (red) and nuclei stained by SYTOX (green) in retinal whole-mounts from wt mice fed normal chow diet and from ApoE^−/−^ mice fed normal chow or 4 weeks of HFD. Bars = 50 µm. Measurements were performed when mice were 17 weeks of age. (**B**) Summarized data from experiments as in **A**, showing increased mean fluorescence intensity of VCAM-1 in ApoE^−/−^ (light gray bar) when compared to wt controls (white bar; ***p<0.001) and further increased VCAM-1 expression in response to HFD (dark gray bar; ^##^ *p*<0.01). (**C**) Mean sVCAM-1 concentrations in plasma from the same mice used in **A** and **B**. ***p<0.001 vs. wt chow and^ #^ *p*<0.05 for differences between ApoE^−/−^ groups (chow vs. HFD). (**D**) Expression of *VCAM-1* mRNA was studied by real time RT-PCR in normolipidemic FVBN mice. Expression was enhanced by 4 or 8 weeks of HFD (plain and patterned gray bars, respectively) when compared to mice fed regular chow diet (white bars). The effects were significant after 8 weeks (*p<0.05). Values are normalized to the expression of cyclophilin B and GAPDH. (**E**) Mean sVCAM-1 concentrations in plasma from the same mice used in **D**. **p<0.01 vs. wt chow.

As shown in **Figure 1**, VCAM-1 protein expression was significantly higher in ApoE^−/−^ than in wt mice when measured at 30 weeks of age. To further elucidate the effect of hyperlipidemia on VCAM-1 expression, we compared expression levels in wt and ApoE^−/−^ mice fed regular chow or high fat diet (HFD), this time at an earlier age of 17 weeks. Confocal immunofluorescence experiments revealed that VCAM-1 protein levels were elevated in the retinal vessels of ApoE^−/−^ mice when compared to wt mice and that expression was further enhanced by HFD (**Figures 5A and B**). As predicted, ApoE^−/−^ mice had higher levels of total cholesterol than wt mice (*p*<0.01); elevated LDL-cholesterol and decreased HDL-cholesterol (*p*<0.01 and *p*<0.001, respectively; **Table 2**). HFD further increased the levels of LDL-cholesterol in ApoE^−/−^ mice (*p*<0.05). A positive correlation was found between VCAM-1 expression and cholesterol (*r* = 0.806, *p*<0.001) and LDL-cholesterol (*r* = 0.796, *p*<0.001), whereas VCAM-1 correlated negatively to HDL-cholesterol (*r* = −0.756, *p*<0.001). VCAM-1 expression did not correlate to plasma triglycerides. In agreement with measurements of sVCAM-1 in plasma from wt and ApoE^−/−^ mice at 30 weeks of age (**Figure 3C**), no differences in sVCAM-1 concentrations were found between wt and ApoE^−/−^ mice at 17 weeks of age, but elevated sVCAM-1 was measured in aged-matched ApoE^−/−^ mice fed HFD for 4 weeks (**Figure 5C**).

In a separate set of experiments, we investigated whether VCAM-1 expression was affected by HFD in normolipidemic mice (ApoE competent). Indeed, *VCAM-1* mRNA measured in isolated retinal vessels revealed higher expression of this adhesion molecule after 8 weeks of HFD (*p*<0.05) and a less pronounced effect after 4 weeks of HFD (**Figure 5D**). After 4 weeks of HFD, elevated blood glucose (*p*<0.05) and decreased plasma triglycerides (*p*<0.05) were observed. Plasma cholesterol was higher both after 4 and 8 weeks of HFD (*p*<0.001, **table 3**). Despite changes in blood glucose and triglycerides, *VCAM-1* mRNA expression correlated only to total plasma cholesterol (*r* = 0.428, *p*<0.05). As for ApoE^−/−^ mice (**Figure 5C**), levels of sVCAM-1 were increased both after 4 and 8 weeks of HFD in these mice (**Figure 5D**). Taken together, these results demonstrate that even moderate changes in plasma cholesterol as those observed in normolipidemic FVBN mice after HFD, are able to drive *VCAM-1* mRNA expression in retinal vessels and are also translated into elevated sVCAM-1 protein in plasma. Interestingly, *ICAM-1* and *E-selectin* mRNA were not affected by HFD (**Figure S2**). No gross morphological changes as assessed by hematoxylin staining were observed in retinal sections of dyslipidemic mice when compared to normolipidemic mice (data not shown).

## Discussion

The present study investigated early retinal endothelial activation in diabetes and/or dyslipidemia by assessment of VCAM-1 expression in mouse retinal vessels, as well as the potential role of TNFα. Our major findings are as follows: (1) VCAM-1 protein levels were increased in retinal vessels of wt mice after 8 weeks of diabetes, at a time-point when the expression of the inflammatory cytokines TNFα, IL-6 and IL-1β was elevated in retina and levels of sVCAM-1 in plasma were higher; (2) TNFα^−/−^ deficient mice exhibited higher basal levels of VCAM-1 protein in retinal vessels and sVCAM-1 in plasma than wt mice, but failed to up-regulate *IL-6* and *IL-1β* mRNA and VCAM-1 protein in response to diabetes. (3) Basal VCAM-1 protein expression in retinal vessels was higher in hyperlipidemic ApoE^−/−^ than in normolipidemic wt mice and both *VCAM-1* mRNA and protein levels were further increased by high fat diet, probably driven by changes in plasma cholesterol, LDL- and HDL-cholesterol, but not in triglycerides; (4) Diabetes had no effects on VCAM-1 protein expression or on plasma sVCAM-1 levels in ApoE^−/−^ mice, but it increased *ICAM-1* mRNA expression in retinal vessels, apparently driven by changes in plasma triglycerides independently of plasma cholesterol.

Our results in wt mice show that STZ-induced hyperglycemia results in enhanced endothelial activation in mouse retinal vessels, as assessed by measurements of VCAM-1 protein expression. This is in line with the proposed mechanism underlying the pathogenesis of diabetic retinopathy, namely that hyperglycemia through various pathways (including accumulation of sorbitol and advanced glycation end-products, oxidative stress, up-regulation of the renin-angiotensisn system and vascular endothelial growth factor) initiates a cascade of events leading to retinal vascular endothelial dysfunction[37]. As we demonstrated in a previous study in cerebral arteries[35], VCAM-1 is not only confined to the endothelium, but it is also expressed in smooth muscle cells surrounding the larger arteries of the retina. The fact that the effect of diabetes on VCAM-1 was predominant in small caliber vessels (<10 µm) is interesting considering that capillary degeneration begins early in diabetes retinopathy and as it advances, contributes to the large non-perfused areas of the retina.

In the retinas of diabetic wt mice, we also found significantly increased levels of *TNFα*, *IL-6* and *IL-1β* mRNA expression and a tendency to increased *IFNγ* and *caspase-1* mRNA, indicating some degree of local inflammation after 8 weeks of diabetes. Plasma levels of these pro-inflammatory cytokines have been suggested as potential biomarkers associated with hyperglycemia and diabetes comorbidities, of value for prediction of diabetes complications[6]. Local elevation of these cytokines in diabetes has indirectly been demonstrated from measurements in the vitreous of patients[38], [39], [40]. Direct experimental evidence in mice models of diabetes is limited, with a study demonstrating elevation of *TNFα* mRNA in male C57Bl6 mice 5 months after induction of diabetes by STZ[41] and another study showing activation of caspase-1/IL-1β signaling in retinas of diabetic mice[42]. Evidence for local up-regulation of *IL-6* and *IFNγ* in retina in diabetes comes from studies in rats[43], [44]. In our hands, diabetic wt mice exhibited higher plasma sVCAM-1, suggesting not only up-regulation of inflammatory mediators in the retina, but also a more systemic endothelial activation in this model.

Another finding in our study was that basal VCAM-1 protein expression in retinal vessels was elevated in ApoE^−/−^ mice compared to wt mice, the difference being more pronounced in younger mice (30 vs. 17 weeks of age), and further elevated by HFD. Induction of VCAM-1 expression by HFD was clear both in normolipidemic FVBN mice after 8 weeks of diet and in hyperlipidemic ApoE^−/−^ already after 4 weeks of diet. One explanation for these differences in basal- and diet-induced VCAM-1 expression could be the differences in plasma cholesterol, HDL- and LDL-cholesterol between genotypes, since these parameters seem to correlate well with VCAM-1 levels in retinal vessels. This cholesterol-sensitivity of VCAM-1 expression we show in retinal vessels is consistent with features of VCAM-1 described in the context of atherosclerosis, where endothelial cells express VCAM-1 in response to cholesterol feeding selectively in areas prone to lesion formation and before leukocyte recruitment begins[11]. Of interest to note is that this cholesterol-sensitivity of VCAM-1 was not shared by ICAM-1 or E-selectin, since at least in our models, HFD had no significant effects on the expression of these adhesion molecules. In the vessel wall, the mechanism of VCAM-1 induction by HFD seems to be dependent on inflammation, initiated by modified lipoprotein particles such as oxidized phospholipids and short-chain aldehydes, which in turn activate VCAM-1 transcription via activation of NF-κB[45]. Interestingly, TNFα and IL-1β have also been shown to induce VCAM-1 expression by this pathway[11] and may account in the absence of hyperlipidemia but under hyperglycemic conditions (such as in the diabetic wt mice in this study), for the observed up-regulation of VCAM-1 in retinal vessels.

Long-term effects of dyslipidemia may be of importance for progression of diabetic retinopathy, as shown by Barile et al.[46], who demonstrated accelerated signs of diabetic retinopathy in hyperglycemic-hyperlipidemic ApoE^−/−^ db/db mice at 6 months of age compared to hyperglycemic-normolipidemic mice. They show that the aggravation of diabetic retinopathy was associated with an increased activation of the advanced glycation end-product and its receptor (AGE/RAGE). Activation of the AGE/RAGE axis has been demonstrated to correlate with increased inflammation and leukocyte recruitment as well as TNFα activity[47], and would provide an alternative link between dyslipidemia, endothelial activation/inflammation and diabetic retinopathy.

Another conclusion from the present study is that measurements of vascular endothelial markers by RT-PCR using total retinal RNA have limited resolution and are not sensitive enough to dissect e.g. the effects of diabetes or genotype on *VCAM-1* expression. Instead, other methods such as confocal microscopy for spatial resolution of VCAM-1 protein and/or RT-PCR for *VCAM-1* mRNA using RNA from isolated retinal vascular networks seem more appropriate. This is best illustrated by the lack of significant differences in the expression of *VCAM-1, ICAM-1* and *E-selectin* mRNA in ApoE^−/−^ mice in response to diabetes when whole retinas were examined, as opposed to a clear increase of *ICAM-1* in response to diabetes when expression was examined in isolated vessels. This diabetes-induced *ICAM-1* expression in ApoE^−/−^ mice was not accompanied by increased levels of pro-inflammatory cytokines in retina, nor with elevated sVCAM levels, suggesting a different scenario than that found in diabetic wt mice. Further, *ICAM-1* mRNA expression correlated to plasma triglyceride but not to cholesterol levels. Collectively, it seems that diet-induced VCAM-1 expression may be driven by serum cholesterol, whereas diabetes-induced ICAM-1 expression may be driven by triglycerides in ApoE^−/−^ mice, by a mechanism yet to be described. Our data also highlights potential differential regulation of adhesion molecules in retinal vessels.

TNFα is a multifunctional cytokine with important roles in inflammation and apoptosis, but also able to exert anti-inflammatory and protective actions[30], [32], depending on receptor type and cellular and environmental status. TNFα has been proposed as a link between metabolic dysregulation and inflammation and/or vascular dysfunction in diabetes[47], [48], and it is well established as a key molecule in diabetic retinopathy[1]. In the present study we found that TNFα^−/−^ deficient mice exhibited higher basal levels of VCAM-1 protein in retinal vessels and sVCAM-1 in plasma than wt mice, but these mice failed to up-regulate *IL-6* and *IL-1β* mRNA and VCAM-1 protein in response to diabetes. These results suggest a dual role for TNFα in the regulation of VCAM-1 expression in retinal vessels, being protective under basal conditions but promoting endothelial activation in response to diabetes. Further, lack of TNFα prevented the up-regulation of *IL-6* and *IL-1β* mRNA otherwise observed in wt mice in response to diabetes. This absence of inflammatory component in the hyperglycemic situation may be the reason why VCAM-1 expression is not increased in the retinal vessels of diabetic TNFα^−/−^ mice. In line with this idea, recent *in vitro* studies using human endothelial cells, demonstrated that hyperglycemia alone was not sufficient to induce expression of VCAM-1, but it significantly enhanced the induction of endothelial VCAM-1 elicited by IL-1β[49]. Results from experiments using TNFα deficient mice should be interpreted with caution, since we cannot exclude a generalized defect in the immune system[31], [32], [50]. However, results from studies using the TNFα inhibitor etanercept on diabetic rats or TNF receptor deficient mice, recently provided strong evidence for the role of TNFα as a promoter of retinal dysfunction in diabetes[51].

In summary, STZ-induced hyperglycemia triggers an inflammatory response in the retina of normolipidemic mice and concomitant up-regulation of VCAM-1 in retinal vessels. Hypercholesterolemia effectively promotes VCAM-1 expression without evident stimulation of inflammation. Diabetes-induced endothelial activation in ApoE^−/−^ mice seems driven by elevated plasma triglycerides and not by cholesterol. Results also suggest a complex role for TNFα in the regulation of VCAM-1 expression, being protective under basal conditions but promoting endothelial activation in response to diabetes. This study also highlights the interplay between inflammatory and metabolic abnormalities leading to retinal endothelial activation in the context of diabetes.

## Materials and Methods

### Mice

All animals were treated according to the Principles for the Care and Use of Animals in Ophthalmic and Vision Research approved by the Association for Research in Vision and Ophthalmology. All experiments were approved by the Malmö/Lund Animal Care and Use Committee. Female C57BL/6 wild-type (wt) and ApoE^−/−^ (B6.129P2-Apoe*^tm1Unc^*N11) mice were purchased from Taconic (Lille Skensved, Denmark) and TNFα^−/−^ (B6, 129-*Tnf^tmlGkl^*) mice from the Jackson Laboratory (Charles River, Sulzfeld, Germany). TNFα^−/−^ and ApoE^−/−^ mice were intercrossed and F7 TNFα^−/−^ and F10 ApoE^−/−^/TNFα^−/−^ progeny were used in the experiments. For the experiments summarized in **Figures 3B** and **5**, and in **Figure S2**, all mice were from Jackson Laboratory (strain name for ApoE^−/−^: B6.129P2-Apoe*^tm1Unc^*/J). For the effects of high fat diet, normolipidemic FVBN mice were also used. Animals had free access to tap water and were fed normal chow (R3; Lantmännen, Kimstad, Sweden) or high fat diet (HFD; R638: 0.15% cholesterol, 21% fat; Lantmännen) as indicated in the text.

### Study design

Data from 3 different sets of *in vivo* experiments are included in this manuscript. In the first one, we investigated the effects of diabetes and genotype on VCAM-1 expression, whereas in the second and third sets, we examined the effects of HFD in dyslipidemic and normolipidemic mice respectively.

In the first set of experiments, wt, ApoE^−/−^, TNFα^−/−^ and ApoE^−/−^/TNFα^−/−^ mice received intraperitoneal injections of streptozocin (STZ; Sigma-Aldrich, Stockholm, Sweden; 60 mg in citrate buffer per kg body weight, pH 4.5) or vehicle (citrate buffer) once a day for 5 days at an age of 22 weeks. Body weight was measured once a week and animals with >15% weight loss were excluded from the study (two C57BL/6 wt, one TNFα^−/−^ and four ApoE^−/−^ mice). Blood glucose was measured once a week in whole venous blood using a One-Touch glucometer (LifeScan Inc., CA, USA*).* 8 weeks after the first STZ injection, animals were anaesthetized with 300 µL intraperitoneal mixture of distilled water, fentanyl-fluanisone and midazolam (2∶1∶1), and euthanized by exsanguination through cardiac puncture. Blood samples were collected and eyes immediately enucleated. Retinas were carefully dissected and either used for immunohistochemistry or for mRNA expression analysis in whole retinas. For immunohistochemistry, retinas were flattened by four radial cuts, mounted on filter paper with the vitreous side up, and thereafter fixated in Histochoice at 4°C. For mRNA analysis, retinas were gently peeled off from the pigment epithelium before frozen on dry ice and stored at −80°C. The numbers of mice included were 28 wt (16 STZ and 12 vehicle), 29 ApoE^−/−^ (17 STZ and 12 vehicle), 24 TNFα^−/−^ (14 STZ and 10 vehicle) and 30 ApoE^−/−^/TNFα^−/−^ (15 STZ and 15 vehicle). For mRNA expression in isolated retinal vessels (see real-time RT-PCR below), additional ApoE^−/−^ mice (12 STZ and 12 vehicle) were used.

For the effects of HFD, two separate sets of animals were used: 1) 7 wt and 14 ApoE^−/−^ mice (7 chow and 7 HFD, 1 month on diet), 13 weeks of age at the start of the experiment and 2) 29 FVBN mice (16 chow and 13 HFD, 1 or 2 months on diet). Retinas from these mice were used for mRNA expression in isolated retinal arteries and for immunohistochemistry of whole mounts. Monitoring of body weight and blood glucose, and termination of the experiment were performed as explained above.

### Extraction of RNA from intact retina

Extraction of total retinal RNA was performed according to a modified Chomczynski protocol[52] as previously described[53]. Each retina was homogenized on a rotor-stator Polytron (PT1200 Kinematica AG, Littau-Lucerne, Switzerland) in 1 mL of TRI reagent (Sigma-Aldrich, Stockholm, Sweden) with 5 µL of Polyacryl carrier (Molecular Research Center, MRC, Cincinnati, OH, USA). After addition of 100 µL of 1-bromo-3-chloropropane (BCP, Sigma-Aldrich, Stockholm, Sweden) samples were vortexed and left for 15 minutes before phases were separated by centrifugation at 12000×g for 15 minutes at 4°C. The aqueous phase (RNA) was precipitated with 500 µL of isopropanol at 12000×g for 10 minutes at 4°C. The pellet was dissolved in 50 µL of DEPC-H_2_O supplemented with 60 U of RNasin Plus RNase Inhibitor (Promega, Madison, USA). Total RNA quantification was performed on a spectrophotometer (Biophotometer, Eppendorf, Hamburg, Germany), after which samples were stored at −80°C until analysis.

### Isolation of retinal vessels and extraction of RNA

Retinal vasculature was selectively isolated from other components of the retina using a modified procedure from the one described by Navaratna[54]. Briefly, retinas were dissected in ice-cold Ca^2+^-free physiological saline solution (PSS, containing in mmol/L: NaCl, 135; KCl, 5,9; MgCl_2_, 1,2; Hepes, 11,6; glucose 2,0; pH 7,4), incubated in ice-cold sterile water for 1 hour at 4°C followed by incubation with bovine pancreatic DNase (25 U, Sigma) for 10 min. Microvascular networks were cleared from debris by gently pipetting with a wide-bore Pasteur pipette.

Extraction of RNA from isolated vessels was performed using a slightly modified protocol as that used for whole retinas. Here, an Omni Tissue Homogenizer (TH International) and 200 µL (instead of 100 µL) of 1-bromo-3-chloropropane were used; samples were vortexed and left for 2–3 minutes before separation of phases. RNA was precipitated with a combination of 500 µL of isopropanol and 2,5 mg Linear acrylamide (Ambion, Texas, USA) and left overnight at −80°C. After a new centrifugation at 12000×g for 15 minutes at 4°C, the pellet was washed in 75% ethanol, further centrifuged and dissolved in 13 µL of DEPC-H_2_O supplemented with 13 U RiboLock RNase inhibitor (Fermentas GMBH, St. Leon-Rot, Germany).

### Real time RT-PCR

For the total retinal RNA analysis, cDNA was synthesized from 2 µg of RNA using 200 U RevertAid RNase H^−^ RT (Fermentas, Helsingborg, Sweden) and 250 ng random hexamer (Amersham Biosciences, Uppsala, Sweden) primer for two hours at 42°C. Expression of VCAM-1, ICAM-1, P-selectin, E-selectin, TNFα, IL-6, IL1β, IFNγ, caspase-1 and the internal control cyclophilin B (Ppib) mRNA levels were analyzed using real-time RT-PCR on a 7900HT system (Applied Biosystems, Stockholm, Sweden). TaqMan assays were from Applied Biosystems (assays on demand); VCAM-1 (Mm01320970_m1), ICAM-1 (Mm00516023_m1), E-selectin (Mm00441278_m1), P-selectin (Mm00441295_m1), TNFα (Mm99999068_m1), IL-6 (Mm00446190_m1), IL-1β (Mm01336189_m1), IFNγ (Mm01168134_m1), Caspase 1 (Mm00438023_m1) and Ppib (Mm00478295_m1). For RNA analysis in isolated retinal vessels, cDNA was synthesized with the RevertAid^TM^ H Minus M-MuLV Reverse transcriptase (Fermentas GMBH, St. Leon-Rot, Germany) according to the manufacturer's instructions. The Gene expression Assays used were: VCAM-1 (Mm01320970_m1), ICAM-1 (Mm00516023_m1), E-selectin (Mm00441278_m1) and Ppib (Mm00478295_m1) and GAPDH (4352339E) as housekeeping controls. The relative quantity of target genes was calculated using the comparative threshold method (ΔΔC_t_) and using Ppib as endogenous control as previously described[55]. For isolated vessels Genormv. 3.5 software was used to relate target expression to both endogenous controls, Ppib and GAPDH.

### Confocal immunofluorescence: VCAM-1 detection

Whole mount retinas were cleaned in phosphate-buffered saline (PBS, pH 7.4; Sigma-Aldrich, Stockholm, Sweden), permeabilized with 0.2% Triton X-100 in PBS and blocked with 2% bovine serum albumin (BSA) in PBS for 2 hours. For detection of VCAM-1 the primary antibody rat anti-mouse CD 106 (#M/K-2; Chemicon International, Inc., Millipore, Solna, Sweden) was diluted 1∶400 in 2% BSA/PBS and applied overnight at 4°C. The secondary antibody, Cy5 anti-rat IgG (Jackson ImmunoResearch Laboratories, Charles River, Sulzfeld, Germany), was diluted 1∶500 and applied for 2 hours at room temperature. For identification of nuclei, the fluorescent nucleic acid dye SYTOX Green (Invitrogen, Paisley, UK) 1∶3000 was applied for 10 minutes. Whole retinas were mounted on slides (Aqua Polymount mounting medium, Polysciences, Eppelheim, Germany), examined at 63X using a Zeiss LSM 5 Pascal laser scanning confocal microscope. VCAM-1 was detected by monitoring Cy5 fluorescence using an excitation wavelength of 633 nm and an emission wavelength of >650 nm. At least 3 images were taken from each vessel branch and approximately 80 images were taken on average per retina. Mean pixel intensity (ranging 0 to 255 grayscale values, after background subtraction) and vessel diameter were measured using the Zeiss LSM 5 software. Specificity of immune staining was confirmed by the absence of fluorescence in arteries incubated with primary or secondary antibodies alone (**Figure S3**). Experiments as well as analysis were performed under blind conditions.

### Total plasma cholesterol, HDL and triglycerides

Total plasma cholesterol and triglycerides were quantified with colorimetric assays, InfinityTM Cholesterol and InfinityTM Triglycerides (both from Thermo Scientific, Middletown, VA, USA) according to the manufacturer's instructions. HDL levels were measured after precipitation of Apo-B containing lipoproteins using a modified version of a previously described protocol[56]. Briefly, plasma was diluted 1∶4 with PBS and VLDL and LDL precipitated by adding Dextran Sulphate Na-salt (1 mg/ml) and MgCl2 (0.09 M), for 1 hour at +4°C. After centrifugation, HDL cholesterol content was examined in the supernatant. Absorbance was measured at 492 nm (Tecan Sunrise, program Magellan). LDL was calculated by the Friedewald equation[57]: LDL  =  total cholesterol – [HDL + (Triglycerides ×0.20)]. All triglyceride values were below 4.52 mmol/l, which is the recommended TG limit for indirect LDL calculations.

### sVCAM-1 ELISA

The levels of sVCAM-1 in plasma were assayed using Quantikine® Mouse sVCAM-1 ELISA kit (R&D Systems, Abingdon, UK) according to the manufacturer's instructions. Absorbance was measured at 450 nm (Tecan Sunrise, program Magellan) and the lower limit of detection was 0.31 ng/ml.

### Statistics

Results are expressed as mean ± SEM unless otherwise stated in the figure legends. Statistical analysis was performed using SPSS version 15.0.1 and Graph Pad software (Prism 4.0). Analyses of distributions were performed before decisions were made to use parametric tests. Statistical significance was determined using Student's t-test, one- or two-way ANOVA as specified in the text, followed by Bonferroni post hoc tests. Pearson's test was used for correlation analyses.

## Supporting Information

Figure S1 (**A**) Confocal immunofluorescence microscopy images showing merged VCAM-1 (red)- and nuclei (green) fluorescence in the left panel; single VCAM-1 fluorescence in the middle panel and single green fluorescence in the right panel. Images are from a retinal whole-mount from a non-diabetic ApoE^−/−^ mouse. Note adjacent VCAM-1 positive and negative vessels (white arrows). Bars = 50 µm. (**B**) Summarized calculations from confocal immunofluorescence microscopy data showing percentage of VCAM-1 positive and VCAM-1 negative vessels in retinas from control non-diabetic and diabetic wt, ApoE^−/−^, TNFα^−/−^ and ApoE^−/−^/TNFα^−/−^ mice. The percentage of VCAM-1 positive vessels was reduced in diabetic animals from wt and ApoE^−/−^ groups (*p<0.05 and ***p<0.001, respectively), but unaltered in the TNFα^−/−^ genotypes.
(7.14 MB TIF)

Figure S2 Expression of *ICAM-1* and *E-selectin* mRNA was studied by real time RT-PCR in normolipidemic FVBN mice. Expression was not affected by 4 or 8 weeks of HFD (plain and patterned gray bars, respectively) when compared to mice fed regular chow diet (white bars). Values are normalized to the expression of cyclophilin B and GAPDH.
(1.29 MB TIF)

Figure S3 Negative control: Representative confocal immunofluorescence microscopy image showing absence of red immunofluorescence in vessels incubated with secondary antibody alone (Cy5 anti-rat IgG). Retinal whole mount was counterstained with SYTOX green for structure identification. Bars = 50 µm.
(6.95 MB TIF)
