# Supplementary figures and images for: Vascular Cellular Adhesion Molecule-1 (VCAM-1) Expression in Mice Retinal Vessels Is Affected by Both Hyperglycemia and Hyperlipidemia
Source: PLoS One. 2010 Sep 13;5(9):e12699. doi: 10.1371/journal.pone.0012699 (PMC2938334; doi:10.1371/journal.pone.0012699)

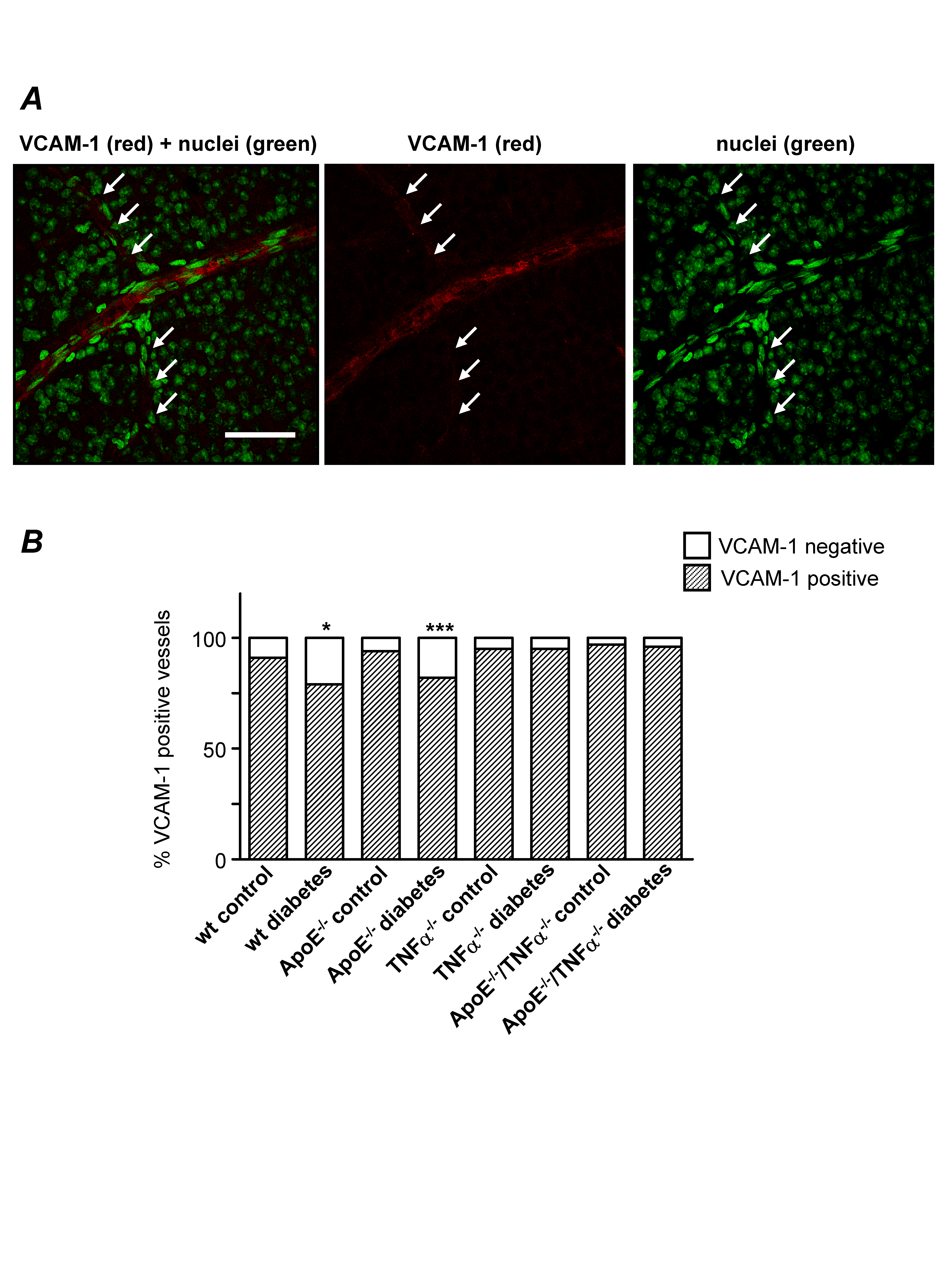

Supplement: Figure S1 — (A) Confocal immunofluorescence microscopy images showing merged VCAM-1 (red)- and nuclei (green) fluorescence in the left panel; single VCAM-1 fluorescence in the middle panel and single green fluorescence in the right panel. Images are from a retinal whole-mount from a non-diabetic ApoE−/− mouse. Note adjacent VCAM-1 positive and negative vessels (white arrows). Bars = 50 µm. (B) Summarized calculations from confocal immunofluorescence microscopy data showing percentage of VCAM-1 positive and VCAM-1 negative vessels in retinas from control non-diabetic and diabetic wt, ApoE−/−, TNFα−/− and ApoE−/−/TNFα−/− mice. The percentage of VCAM-1 positive vessels was reduced in diabetic animals from wt and ApoE−/− groups (*p<0.05 and ***p<0.001, respectively), but unaltered in the TNFα−/− genotypes. (7.14 MB TIF) [file pone.0012699.s001.tif]

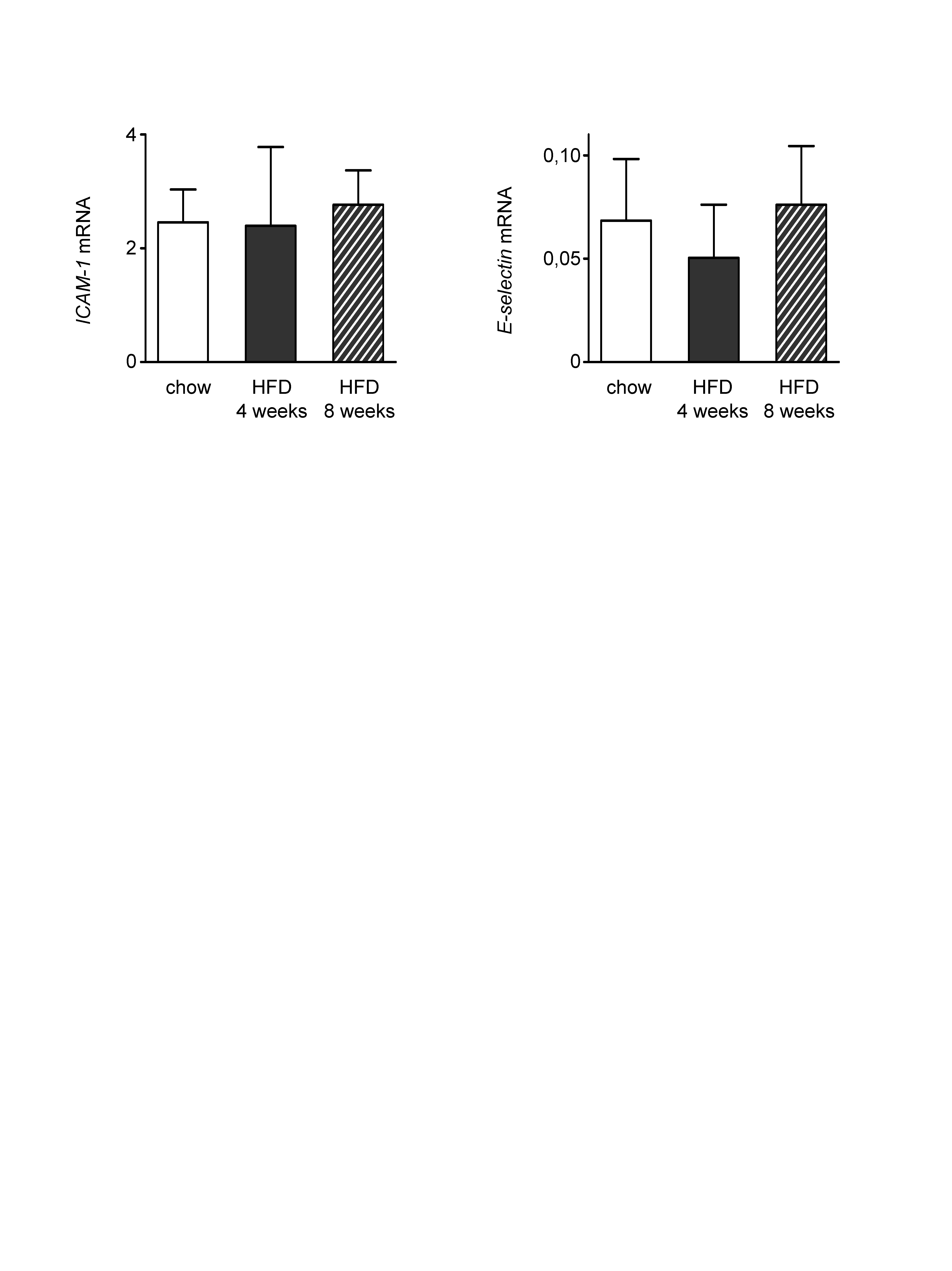

Supplement: Figure S2 — Expression of ICAM-1 and E-selectin mRNA was studied by real time RT-PCR in normolipidemic FVBN mice. Expression was not affected by 4 or 8 weeks of HFD (plain and patterned gray bars, respectively) when compared to mice fed regular chow diet (white bars). Values are normalized to the expression of cyclophilin B and GAPDH. (1.29 MB TIF) [file pone.0012699.s002.tif]

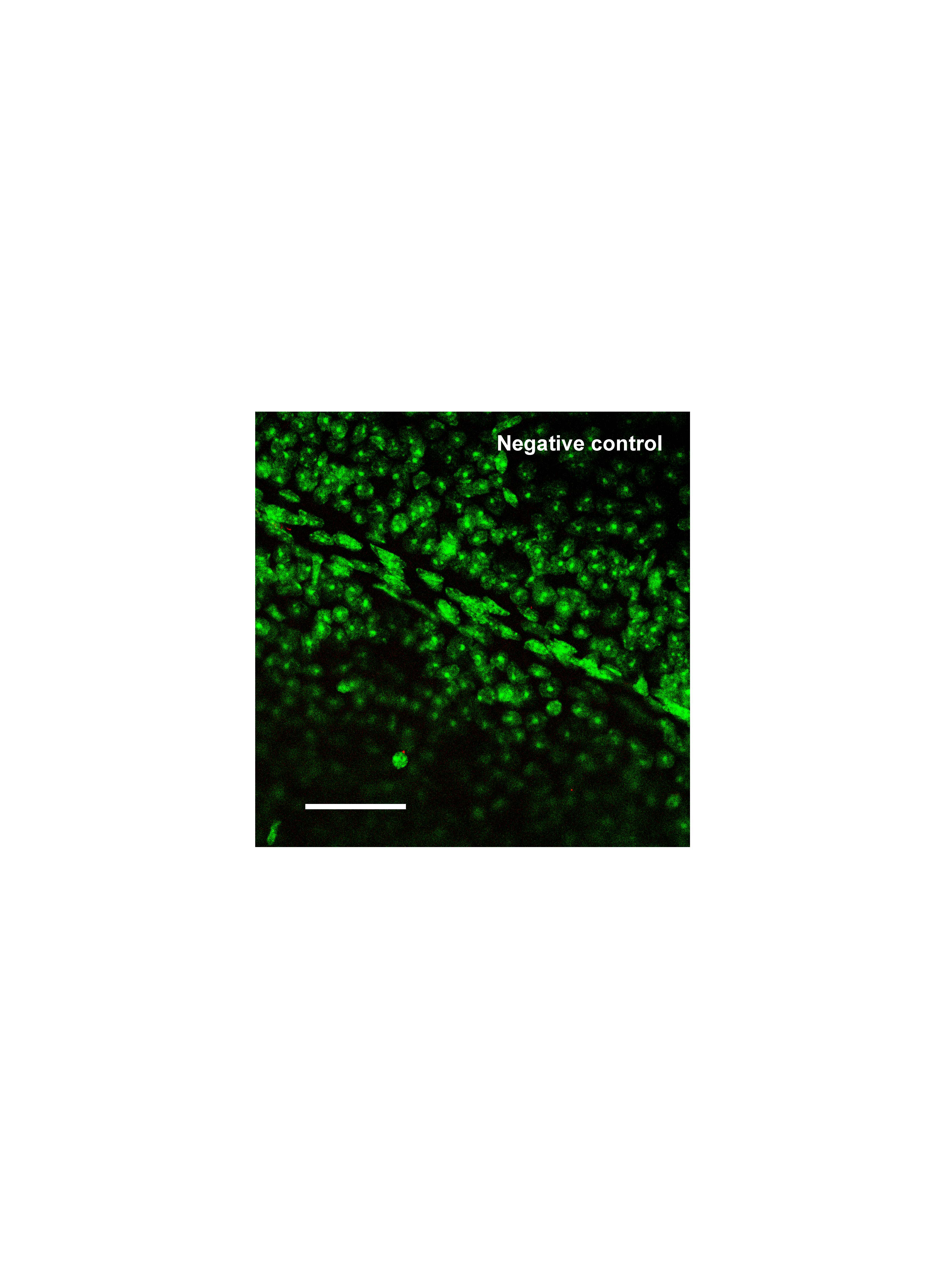

Supplement: Figure S3 — Negative control: Representative confocal immunofluorescence microscopy image showing absence of red immunofluorescence in vessels incubated with secondary antibody alone (Cy5 anti-rat IgG). Retinal whole mount was counterstained with SYTOX green for structure identification. Bars = 50 µm. (6.95 MB TIF) [file pone.0012699.s003.tif]
